# Diagnosis of Amnesic Mild Cognitive Impairment Using MGS-WBC and VGBN-LM Algorithms

**DOI:** 10.3389/fnagi.2022.893250

**Published:** 2022-05-30

**Authors:** Chunting Cai, Jiangsheng Cao, Chenhui Yang, E. Chen

**Affiliations:** ^1^School of Informatics, Xiamen University, Xiamen, China; ^2^Department of Neurology, Zhongshan Hospital Affiliated to Xiamen University, Xiamen, China

**Keywords:** machine learning, aMCI, MGS-WBC, multi-scale features, VGBN-LM

## Abstract

Computer-aided diagnosis (CAD) has undergone rapid development with the advent of advanced neuroimaging and machine learning methods. Nevertheless, how to extract discriminative features from the limited and high-dimensional data is not ideal, especially for amnesic mild cognitive impairment (aMCI) data based on resting-state functional magnetic resonance imaging (rs-fMRI). Furthermore, a robust and reliable system for aMCI detection is conducive to timely detecting and screening subjects at a high risk of Alzheimer’s disease (AD). In this scenario, we first develop the mask generation strategy based on within-class and between-class criterion (MGS-WBC), which primarily aims at reducing data redundancy and excavating multiscale features of the brain. Concurrently, vector generation for brain networks based on Laplacian matrix (VGBN-LM) is presented to obtain the global features of the functional network. Finally, all multiscale features are fused to further improve the diagnostic performance of aMCI. Typical classifiers for small data learning, such as naive Bayesian (NB), linear discriminant analysis (LDA), logistic regression (LR), and support vector machines (SVMs), are adopted to evaluate the diagnostic performance of aMCI. This study helps to reveal discriminative neuroimaging features, and outperforms the state-of-the-art methods, providing new insights for the intelligent construction of CAD system of aMCI.

## Introduction

Alzheimer’s disease (AD), which occurs frequently in elderly individuals, is a chronic and irreversible neurodegenerative disease accompanied by brain impairments in memory, communication, and reasoning (Dadar et al., 2017). The new report indicates that more than 5.7 million individuals have been diagnosed with AD in the United States (Association, 2018). Unfortunately, as yet, there is no agreed medication or treatment protocol to cure and rehabilitate patients with AD (Xi et al., 2022). Amnesic mild cognitive impairment (aMCI), which is generally characterized by prominent deficits in memory, is widely considered as the early stage of AD due to its greater risk of conversion to AD (Bian et al., 2014; Barone et al., 2016). Accordingly, a robust and reliable system for aMCI detection is conducive to timely detecting and screening the subjects with AD at high risk, thus providing an optimal treatment period for patients.

Resting-state functional magnetic resonance imaging (rs-fMRI), which reflects the neural functional activity of the brain by measuring spontaneous blood oxygen level dependency (BOLD) signal fluctuations in a non-invasive way, has been widely employed as one of the important techniques to diagnose brain-related diseases (Li et al., 2018; Li R. et al., 2020; Sundaram et al., 2020). However, rs-fMRI data are confronted with formidable challenges to the existing pattern classification methods due to its limited data samples and high dimensions, which is not favorable for aMCI detection (Kassani et al., 2020). In consequence, existing methods for observing alterations of spontaneous neural activity, such as regional homogeneity (ReHo), the amplitude of low-frequency fluctuation (ALFF), and functional connectivity analysis, are employed to explore the biological mechanisms of brain function and reduce the redundant information of brain (Harrison et al., 2019; Liao et al., 2019; Ting et al., 2020; Xiao et al., 2020). Furthermore, the aMCI can be diagnosed using rs-fMRI data processed by the use of abovementioned methods as reported in a few studies (Zhou et al., 2014; Kim et al., 2020; Li Y. et al., 2020). Consequently, the diagnosis of aMCI based on these data can be employed for timely prevention and screening of potential patients with AD.

In this study, the data sample after preprocessing is then conducted using ReHo calculation, ALFF calculation, and functional connectivity construction, and we obtain two three-dimensional whole-brain structures and the brain functional network. Then, we develop an MGS-WBC model, which mainly consists of the intraclass volatility detection method using variable coefficient and multiscale statistical thresholds, to excavate the multiscale features and reduce the redundancy information both in the whole-brain structures and functional network. It is remarkable that the traditional approaches only use the single local features of the functional network as the input of the classifier, while ignoring its global features. Therefore, we present a VGBN-LM model that extracts the global features and then concatenates local features obtained from the MGS-WBC model to further excavate features of the functional network. Then, we fuse the features that resulted from the MGS-WBC model and VGBN-LM model to further improve the diagnostic performance of aMCI. Concurrently, to evaluate the validity of obtained features and diagnostic performance of aMCI, typical machine learning classifiers applied to the limited number of data, such as naive Bayesian (NB), linear discriminant analysis (LDA), logistic regression (LR), and support vector machines (SVMs), are utilized. Noting that the leave-one-out cross-validation is employed to avert the overfitting problem in this study (Sangnawakij and Niwitpong, 2017). This study greatly improves the diagnostic performance of aMCI compared with the traditional methods, providing new insights for the construction of the computer-aided diagnosis (CAD) system for neurodegenerative diseases, especially for aMCI.

Herein, it is not ideal how to extract discriminative features from the limited and high-dimensional data, especially for aMCI data based on rs-fMRI. Furthermore, a robust and reliable system for aMCI detection is conducive to timely detecting and screening subjects at a high risk of AD. Hence, the novel aspect of this study is that we first develop an MGS-WBC model to extract discriminative features of whole-brain structures and functional networks using volatility detection and multiple significant thresholds. In the following section, we present a VGBN-LM model that excavates the global features of the functional network and fuses its local features, thus overcoming the low availability of the network feature. Finally, all features generated from the MGS-WBC model and VGBN-LM model are concatenated to further improve the diagnostic performance of aMCI.

The remainder of the study is structured as follows: We discuss the related studies on feature extraction and the diagnosis of aMCI in the “Related Work” Section. We present data source, data acquisition, and data preprocessing in the “Materials” Section. We describe the methodologies of aMCI’s diagnostic system in the “Methods” Section. We provide the experiment results and analysis in the subsequent section and give the conclusion in the final section.

## Related Work

Advanced neuroimaging, machine learning techniques, and statistical algorithms provide an opportunity to understand how the brain works between healthy control (HC) and patients with brain diseases, and to analyze the pathological mechanism of the brain (Nadarajah and Kotz, 2006; Lei et al., 2019). Additionally, rs-fMRI, which is convenient and suitable for patients with cognitive impairment who feel difficult to complete tasks, is widely employed in the study of diseases related to the central nervous system, which can reflect the functional condition of the brain under the default state. Nevertheless, how to extract discriminative features from the limited and high-dimensional data is not ideal, especially for the aMCI data based on rs-fMRI.

To address this issue, numerous previous studies have focused on excavating biomarkers associated with aMCI disease from the perspective of neuroimaging. For example, compared with the HC group, abnormal patterns of diverse-club and rich-club organizations in the functional network are revealed using the two-sample t-test in the aMCI group, indicating that the overlapping nodes might be potential biomarkers in the diagnosis of aMCI (Xue et al., 2020). Also, a previous study has revealed that abnormal alterations of ALFF in patients with aMCI are found involved in brain regions, such as the right hippocampus, parahippocampal cortex, and left lateral temporal cortex, which may act as biomarkers of disease (Xi et al., 2013). The former investigation has also confirmed that disruptive patterns of ReHo are found using the two-sample *t*-test in the brain, which is conducive to better comprehending the neural substrates of aMCI and can be served as biomarkers (Zhen et al., 2018). It should be noted that all the studies mentioned above are conducted using statistical methods and existing medical background knowledge to analyze the disruptive patterns of brain disease, ignoring the adoption of machine learning techniques to assess the validity and reliability of diagnostic results.

Currently, machine learning techniques have been actively adopted by researchers to design the CAD system of aMCI, which mainly aims at improving the classification performance and putting it into clinical practice (Zhang et al., 2015; Yang et al., 2021). To the limited number of data and information redundancy problems, feature selection is first implemented and then acted as the input of machine learning classifiers, which are suitable for small data learning, such as NB, LDA, LR, and SVM. A previous investigation based on the functional network classification of aMCI has elucidated that the obvious brain regions which resulted from the two-sample t-test are served as the input of SVM classifier, resulting in the ACC of 69% (Lee et al., 2015). Moreover, it has been reported that the identification ACC of aMCI is 75.35% when using significant regions of the ALFF as the input of SVM classifier (Yang et al., 2018). Up to now, the existing literature not only lacks quantity in the concerned study but also focuses on the single significant threshold method, ignoring the extraction of multiscale features in the brain.

In this study, we first develop an MGS-WBC model using validation detection and multiple significant thresholds methods to extract multiscale features both in the whole-brain structures and functional network. Concurrently, we present the VGBN-LM model to extract the global features of the functional network and fuse its local features. Finally, we fuse the extracted features generated from the MGS-WBC model and VGBN-LM model to further improve the diagnostic performance of aMCI. Compared with the traditional method, the results elucidate that the proposed methods provide the accuracy (ACC) of 89.55, 91.04, 92.54, and 94.03% with the same input data in four typical classifiers, including NB, LDA, LR, and SVM, and maximum improvements are 1.49, 1.49, 1.50, and 4.48%, respectively. Moreover, the area under the curve (AUC) on the four sequence classifiers mentioned above are 95.63, 92.69, 97.59, and 97.33%, increasing by 2.49, 1.96, 2.67, and 2.23% compared with the traditional method. It can be inferred that our aMCI diagnostic system is more suitable and reliable than the traditional method. In addition, this study can help reveal impressible and discriminative neuroimaging features, providing new insights for the construction of the CAD system of aMCI.

## Materials

### Description of the Data Source

In this study, the raw data samples are composed of two parts, including 39 patients with aMCI and 38 HCs. These raw data samples are obtained from the second stage of Alzheimer’s Disease Neuroimaging Initiative (ADNI) database, which helps to explore neurological biomarkers of cognitive disease and assist in the diagnosis and treatment of the disease^1^.

It deserves to be further mentioned that not all data samples are conformed to work demands. One data sample has been precluded due to undue head movement (cumulative translation or rotation >2 mm or 2°) and poor quality of image registration (Wang et al., 2018). Concurrently, we exclude 6 patients with aMCI and 4 HCs for inconsistent voxel size or dimension of the brain. To sum up, we end up with 33 patients with aMCI and 34 HCs in this study (Wang et al., 2020).

### Data Acquisition and Preprocessing

All the data samples we focus on in this study are based on rs-fMRI obtained using a clinical 3.0-Tesla (T) scanner. Noting that each raw data sample based on rs-fMRI is composed of brains at 140 points in time. To maintain the reliability of the raw data samples, the subjects are instructed to lay flat, to close their eyes without thinking, and to keep their head in position throughout the imaging processing. We perform the scanner parameters as follows (Li W. et al., 2020): Echo time (TE) = 30 ms, repetition time (TR) = 3,000 ms, voxel size = 3.31 mm × 3.31 mm × 3.31 mm, flip angle (FA) = 80°. More detailed information about scanner parameters based on rs-fMRI data can be inquired on the ADNI’s website.

We perform raw data sample preprocessing using Resting-State fMRI Data Analysis Toolkit plus (RESTplus)^2^, which is based on math software MATLAB2012a^3^ and Statistical Parametric Mapping software (SPM12)^4^. The raw data preprocessing steps we adopt are depicted as follows: first, owing to the machine and human factors, the imaging signal acquired at the beginning of scanning may exhibit unstable signal ingredient factors. Thus, the first 5 time points of each data sample after imaging are discarded in this study. Also, we calibrate the brains of the remaining time points to eliminate the effects induced by discrepancies between various brains or slices. We further normalize the brains of all data samples using the echo-plane imaging (EPI) template from the original coordinate system to Montreal Neurological Institute (MNI) coordinate system due to the shape of each subject’s brain being inconsistent. Specifically, we smooth out the noise that exists in the brain using a Gaussian kernel of 6-mm full width at half maximum (FWHW) for functional network and ALFF analysis (Yang et al., 2021). After that, we remove the variables that affect the dependent variables, including nuisance cerebrospinal fluid signal, global mean signal, white matter signal, and 6 head motion parameters. Finally, the signals with important physiology meaning are obtained through a bandpass filter of 0.01–0.08 Hz (Vicente et al., 2018). Based on the steps mentioned above, the data samples after preprocessing are utilized in the following section.

## Methods

### Overview of the Amnesic Mild Cognitive Impairment Diagnosis System

In this study, we develop a diagnosis system for aMCI. At first, the raw data samples based on the rs-fMRI are pre-processed as depicted in the “Related Work” Section. In the next step, the data samples after preprocessing are calculated using typical approaches used to observe alterations of neuronal activity, such as ReHo, ALFF, and functional connectivity, and we obtain two whole-brain structures and one functional network for each data sample. Then, the calculated data samples are acted as the input of MGS-WBC model and VGBN-LM model we propose to extract biological features of whole-brain structures and functional networks. To evaluate the validity of obtained features, NB, LDA, LR, and SVM are utilized in this study. Furthermore, the leave-one-out cross-validation is carried out to avert the overfitting problem in this study.

**FIGURE 1:**
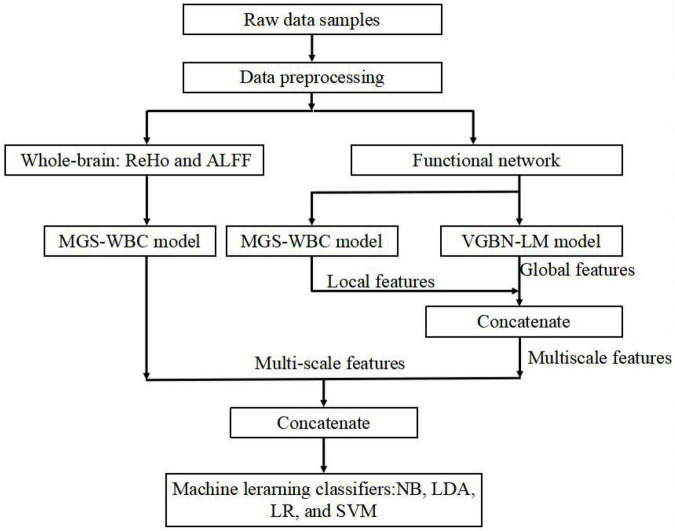
Overall framework of amnesic mild cognitive impairment (aMCI) diagnosis system.

In the results, the features generated by the MGS-WBC model and VGBN-LM model yield better performance than the traditional method in the aMCI detection process. The framework of our aMCI diagnosis system is illustrated in Figure 1.

### Mask Generation Strategy Based on Within-Class and Between-Class Criterion

**FIGURE 2:**
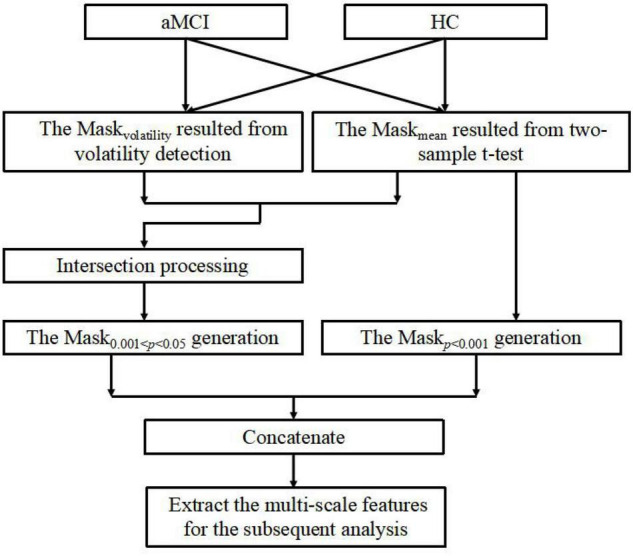
The description of mask generation strategy based on within-class and between-class criterion (MGS-WBC) model.

In this section, we develop an MGS-WBC model to reduce the redundancy both in the whole-brain structures and functional networks and to further excavate the corresponding features of multiscale significant thresholds. Remarkably, the connectivity of the functional network is constructed using the Pearson correlation coefficient (PCC), and the inputs of PCC come from the time series of the corresponding brain regions. Also, to better extract the time series of the brain, we employ the general template of anatomical automatic labeling (AAL) to segment the brain into 90 regions (Ju et al., 2019). The structure of the MGS-WBC model shown in Figure 2 mainly consists of within-class volatility detection using variable coefficient and multi-scale significant thresholds. Since the mask size using a single significant threshold of 0.001 < *p* < 0.05 is much larger than *p* < 0.001, the traditional method only uses the mask with *p* < 0.001 for feature extraction and ignores the effective utilization of features. Besides, for the limited amount of data with a high dimension, how to better extract discriminative features effectively corresponding to the significant threshold of 0.001 < *p* < 0.05 is no clear. First, intraclass volatility detection mask of the whole-brain structures and 90 × 90 functional network, which is defined as Mask_*volatility*_, are generated using variable coefficient within the group to extract the smallest values of the first 5% (Xu et al., 2017), and the variable coefficient (VC) is defined as follows:


(1) $$VC=\frac{\sqrt{\frac{\sum_{i=1}^{n}{\left(x_{i}-\overset{-}{x}\right)}^{2}}{n}}}{\overset{-}{x}}\times 100\%$$

where $\bar{x}$ denotes the average value of the selected pixel or selected brain region, *n* denotes the number of subjects within the group, and *x*_*j*_ denotes the value of the selected pixel or selected brain region corresponding to the ith subject. To obtain the features under multiple significant thresholds, we use Mask_volatility_ and a two-sample *t*-test to generate the mask of 0.001 < *p* < 0.05, defined as Mask_0.001 < *p* < 0.05_, which makes a large difference between the averages of the classes and the fluctuations within the classes are stable. Next, we further use the traditional method that extracts the features obtained from *p* < 0.001 to generate a mask, defined as Mask_*p* < 0.001_. And finally, Mask_0.001 < *p* < 0.05_ and Mask_*p* < 0.001_ are concatenated as the input mask of whole-brain structures or functional networks to extract the final features. It is worth mentioning that the whole-brain structures employed in this study are calculated using the ALFF method and ReHo method, respectively, which are commonly employed to analyze neural activity in the brain and reduce the dimensions of brain data.

### Vector Generation for Brain Networks Based on Laplacian Matrix

In this section, we present a VGBN-LM model for extracting the global features of the functional network. Laplacian Eigenmaps (LE) is a non-linear dimensionality reduction method, which constructs the relationship between samples from the local point of view, and can reflect the local relationship of samples to a certain extent. That is, the points related to each other are expected to be as close as possible to each other in the space after dimensionality reduction. Accordingly, we mainly use LE’s conception to extract global features of the functional network. Here is a brief introduction to the LE algorithm (Belkin and Niyogi, 2001):

Step 1: The construction of the graph is defined as *G*(*V*, *W*): Given *n* samples *x*_1_, *x*_2_…, *x*_*n*_ in *R^d^*, the edge connection *w*_*ij*_ between sample *i* and sample *j* is established using *b* nearest neighbors, and the *w*_*ij*_ can usually be constructed using Gaussian kernel function shown in (2). Notably, *w*_*ij*_ represents symmetric, *D* represents the node degree matrix shown in (3), and *x*_*n*_ represents the eigenvector corresponding to the sample *n*.


(2) $$w_{ij}=e^{-\frac{{||x_{i}-x_{j}||}^{2}}{t}}$$

(3) $$D_{ii}=\sum_{j}w_{ij}$$

Step 2: To maintain the identical geometric property as possible after reducing the dimension of the functional network, it can be solved as the minimization problem of *y^T^Ly*. That is, if the connection value *w*_*ij*_ is larger, the values of *y*_*i*_ and *y*_*j*_ will be closer, as shown in (4). Thus, this reduces now to (5).


(4) $$\begin{array}{c} y^{T}Ly=y^{T}\left(D-W\right)y\\ \;=y^{T}Dy-y^{T}Wy\\ \;=\sum_{i=1}^{n}d_{i}y_{i}^{2}-\sum_{i,j=1}^{n}y_{i}y_{j}w_{ij}\\ \;=\frac{1}{2}\left(\sum_{i=1}^{n}d_{i}y_{i}^{2}-2\sum_{i,j=1}^{n}y_{i}y_{j}w_{ij}+\sum_{j=1}^{n}d_{j}y_{j}^{2}\right)\\ \;=\frac{1}{2}\sum_{i,j=1}^{n}w_{ij}{\left(y_{i}-y_{j}\right)}^{2}\geq 0 \end{array}$$

(5) $$Y_{opt}=\arg\min_{Y^{T}DY=I}tr\left(Y^{T}LY\right)$$

Step 3: Finally, after using the Lagrange multiplier method, the optimal result can be obtained by (6).


(6) Ly=λDy

However, the goal of the LE algorithm is to reduce the dimension of the sample features, and the dimension of the reduced features depends on the number of minimum non-zero eigenvalues, which does not conform to the requirements of extracting the global features from the functional network in this study. Moreover, it is unable to transform the functional network from two dimensions to one dimension that maintains the global features. Thus, considering the limited and high-dimensional data, the VGBN-LM model based on the Laplacian matrix is proposed to reduce the dimension of 90 × 90 functional network and obtain the global features of functional network. Concurrently, we expect that the functional network after dimension reduction operation can maintain and reflect the identical geometric property of functional network. That is, we represent the global features of 90 × 90 functional network in terms of reduced dimension vector.

First, we construct the minimization problem based on the Laplacian matrix. More specifically, let


(7) $$\begin{array}{c} \min y^{T}Ly=\frac{1}{2}\sum_{i,j}w_{ij}{\left(y_{i}-y_{j}\right)}^{2}\\ s.t.y^{T}Dy=\sigma \end{array}$$

where σ represents the special real number and its value will be introduced later. We then use Lagrange to acquire optimal result, which can be formulated as follows:


(8) $$G\left(y,\lambda \right)=y^{T}Ly+\lambda \left(\sigma -y^{T}Dy\right)$$

Then, we take the derivative of *y* and λ of *G*(*y*, λ), respectively.


(9) $$\begin{array}{c} \frac{\partial G\left(y,\lambda \right)}{y}=\frac{\partial \left(y^{T}Ly+\lambda \left(\sigma -y^{T}Dy\right)\right)}{y}\\ \;=\frac{\partial \left(y^{T}\left(L-\lambda D\right)y+\lambda \partial \right)}{y}\\ \;=0\\ \Rightarrow Ly=\lambda Dy \end{array}$$

(10) $$\begin{array}{c} \frac{\partial G\left(y,\lambda \right)}{\lambda }=\frac{\partial \left(y^{T}Ly+\lambda \left(\sigma -y^{T}Dy\right)\right)}{\lambda }\\ \Rightarrow y^{T}Dy=\sigma \end{array}$$

Thus, the optimal result turns to determine the solution of (11).


(11) $$\left\{ \begin{array}{c} Ly=\lambda Dy\\ y^{T}Dy=\sigma \end{array}\right.$$

Nevertheless, the LE algorithm employs constraint conditions of *y^T^Dy* = 1 to obtain the eigenvectors corresponding to all non-zero eigenvalues, ignoring that all the optimal results may not satisfy the original constraint condition. Moreover, the generated eigenvectors of 90 × 90 functional network cannot present its global features due to the excessive redundant features and limited amount of data.

Next, we deform *Ly* = λ*Dy* to obtain the optimal result, and its expression could be written as:


(12) $$\begin{array}{c} Ly=\lambda Dy\\ \Leftrightarrow \left(D-W\right)y=\lambda Dy\\ \Leftrightarrow Dy-\lambda Dy=Wy\\ \Leftrightarrow \left(1-\lambda \right)Dy=Wy\\ \Leftrightarrow y^{T}\left(1-\lambda \right)Dy=y^{T}Wy\\ \Leftrightarrow \left(1-\lambda \right)y^{T}Dy=y^{T}Wy \end{array}$$

If λ ≠ 1, we obtain


(13) $$\begin{array}{c} \left(1-\lambda \right)y^{T}Dy=y^{T}Wy\\ \Rightarrow y^{T}Dy=\frac{y^{T}Wy}{1-\lambda } \end{array}$$

Then, if λ = 1, it can be inferred as below:


(14) $$\begin{array}{c} \left(1-\lambda \right)y^{T}Dy=y^{T}Wy\\ \Leftrightarrow Ly=\lambda Dy\\ \overset{\lambda =1}{⟶}Ly=Dy\\ \Leftrightarrow Wy=0 \end{array}$$

Therefore, the optimal result of (11) can be further deformed as:


(15) $$\left\{ \begin{array}{c} y^{T}Dy=\sigma \\ Ly=\lambda Dy \end{array}\right.\Rightarrow \left\{ \begin{array}{c} \left\{ \begin{array}{c} y^{T}Dy=\frac{y^{T}Wy}{1-\lambda }\\ y^{T}Dy=\sigma \end{array}\right.,if\lambda \neq 1\\ \left\{ \begin{array}{c} Dy=Ly\Leftrightarrow Wy=0\\ y^{T}Dy=\sigma \end{array}\right.if\lambda =1 \end{array}\right.$$

Significantly, the solution of (15) is reached by use of the backward induction. Let (λ_*ξ*_*i*__, *y*_*ξ*_*i*__) represents the solutions of $\left\{ \begin{array}{c} y^{T}Dy=\frac{y^{T}Wy}{1-\lambda }\Leftrightarrow Ly=\lambda Dy\\ y^{T}Dy=\sigma \end{array}\right.if\lambda \neq 1$, then we can deduce that $\sigma_{\xi_{i}}=y_{\xi_{i}}^{T}Dy_{\xi_{i}}$. Based on this, we can obtain the following

form:


(16) $$\begin{array}{c} \arg\min_{y^{T}Dy=\sigma }y^{T}Ly\overset{L\lambda_{\xi_{i}}=\lambda_{\xi_{i}}Dy_{\xi_{i}},y=y_{\xi_{i}}}{⟶}\min\lambda_{\xi_{i}}y_{\xi_{i}}^{T}Dy_{\xi_{i}}\\ \overset{y_{\xi_{i}}^{T}Dy_{\xi_{i}}=\frac{y_{\xi_{i}}^{T}Wy_{\xi_{i}}}{1-\lambda_{\xi_{i}}}}{⟶}\min\lambda_{\xi_{i}}\frac{y_{\xi_{i}}^{T}Wy_{\xi_{i}}}{1-\lambda_{\xi_{i}}}\\ =\min\left(\lambda_{\xi_{1}}\frac{y_{\xi_{1}}^{T}Wy_{\xi_{1}}}{1-\lambda_{\xi_{1}}},\lambda_{\xi_{2}}\frac{y_{\xi_{2}}^{T}Wy_{\xi_{2}}}{1-\lambda_{\xi_{2}}},\ldots ,\lambda_{\xi_{i}}\frac{y_{\xi_{i}}^{T}Wy_{\xi_{i}}}{1-\lambda_{\xi_{i}}}\right)\\ ,i\in \left(1,2,\ldots ,d\right) \end{array}$$

where *d* represents the number of solutions in *Ly* = λ*Dy*. Concurrently, we assume that *Dy = Ly* have the solutions, defined as *y*_β_*j*__, and then we make $\sigma_{\beta_{j}}=y_{\beta_{j}}^{T}Dy_{\beta_{j}}$. From this, it can be deduced that the equation $\left\{ \begin{array}{l} Dy=Ly\Leftrightarrow Wy=0\\ y^{T}Dy=\text{σ} \end{array}\right.if\text{ λ}=1$ have the solution. Therefore, we can deduce the expression as follows:


(17) $$\begin{array}{c} \arg\min_{y^{T}Dy=\sigma }y^{T}Ly\overset{y=y_{\beta_{j}}}{⟶}\min y_{\beta_{j}}^{T}Ly_{\beta_{j}}\\ =\min\left(y_{\beta_{1}}^{T}Ly_{\beta_{1}},y_{\beta_{2}}^{T}Ly_{\beta_{2}},\ldots ,y_{\beta_{j}}^{T}Ly_{\beta_{j}}\right),j\in \left(1,2,\ldots ,h\right) \end{array}$$

where *h* represents the number of solutions in *Dy = Ly*. To sum up, the optimal result of (7) can be written as follows:


(18) $$\begin{array}{c} \arg\min_{y^{T}Dy=\sigma }y^{T}Ly=\min\left(\min\lambda_{\xi_{i}}\frac{y_{\xi_{i}}^{T}Wy_{\xi_{i}}}{1-\lambda_{\xi_{i}}},\min y_{\beta_{j}}^{T}Dy_{\beta_{j}}\right)\\ ,i\in \left(1,2,\ldots d\right)andj\in \left(1,2,\ldots ,h\right) \end{array}$$

Remarkably, the value of σ is obtained from the optimal solution in (18). Here, the procedure of VGBN-LM model can be summarized as follows:

Step 1: Given *n* brain regions extracted using AAL template, we use the absolute value of PCC shown in (19) to construct the edge connection *w*_*ij*_ between brain region *i* and brain region *j* instead of Gaussian kernel function and *b* nearest neighbors method.


(19) $$w_{ij}=\left|\frac{\sum_{i=1}^{n}\left(x_{i}-\overset{\_}{x}\right)\left(y_{i}-\overset{\_}{y}\right)}{\sqrt{\sum_{i=1}^{n}{\left(x_{i}-\overset{\_}{x}\right)}^{2}}\sqrt{\sum_{i=1}^{n}{\left(y_{i}-\overset{\_}{y}\right)}^{2}}}\right|$$

where *x*_*i*_ and *y*_*i*_ denote the two signals from two different brain regions, $\bar{x}$ denotes the average value corresponding to *x*_*i*_, $\bar{y}$ denotes the average value corresponding to *y*_*i*_, and *n* denotes the total number of brain regions in AAL template.

Step 2: We present (7–19) to reduce the dimension of the matrix *w*_*ij*_ from two dimensions to one dimension. Meanwhile, we maintain and reflect the global structure information in the lower dimension space to a certain degree. That is, the closer the connections between brain regions before dimension reduction, the nearer the corresponding values after dimension reduction. In this way, our generated vector *y*_*opt*_resulted from (18) contains global features of functional network.

Step 3: Due to the limited amount of data, the two-sample *t*-test method is employed to select the global features with obvious differences (*p* < 0.05) in the generated vector *y*_*opt*_, and then, we concatenate the local features obtained from MGS-WBC model for the following analysis.

### Multiscale Feature Fusion

We concatenate all the generated features obtained from MGS-WBC model and VGBN-LM model to further improve the diagnostic performance of aMCI. That is, if the dimensions of two input features *x*_*input*_ and *y*_*input*_ are *p* and *q*, then the dimension of the output feature *z* is equal to *p* + *q*.

### Classification Using Naive Bayesian, Linear Discriminant Analysis, Logistic Regression, and Support Vector Machine

As can be seen from the above data, we have obtained multiscale features of whole-brain structures and functional networks using the MGS-WBC model and VGBN-LM mode. Considering the limited amount of data, the selection of an appropriate classifier adopted in this study is crucial to evaluate the validation of obtained features and improve the diagnostic performance of aMCI. Fortunately, the SVM classifier and the LR classifier exhibit effective diagnostic performance in terms of mild cognitive impairment (MCI), and it has been exploited by the majority of researchers (Ciulli et al., 2016; Khatun et al., 2019). In addition, typical classifiers applied to small data learning, such as NB classifier and LDA classifier, are also employed to better assess the generalization ability of features. The following is a brief introduction to the classifiers mentioned above.

The NB classifier mainly uses the probability of known data to determine the classification of the unknown data (Liu et al., 2016):

(1) Each data sample *x* is composed of m-dimensional features, denoted by *a*_1_, *a*_2_, *a*_3_, …, *a*_*m*_.

(2) We calculate the conditional probability for each category under each data sample. The category set is denoted as *C* = {*l*_1_, *l*_2_, …, *l*_*k*_}, hence, we calculate *p*(*l*_1_|*x*), *p*(*l*_2_|*x*), …, *p*(*l*_*k*_|*x*) separately.


(20) $$p\left(l_{k}|x\right)=\frac{p\left(l_{k}\right)p\left(x|l_{k}\right)}{p\left(x\right)}$$

where *p*(*l*_*k*_|*x*) can be deduced by (20) and *k* is the class number of *x*.

(3) If *p*(*l*_*f*_|*x*) = max(*p*(*l*_1_|*x*), *p*(*l*_1_|*x*), …, *p*(*l*_*k*_|*x*)), then *l*_*f*_ is the category corresponding to *x*, where *f*∈(1,2, …, *k*).

The main idea of the LDA classifier is to ensure that the intraclass variance of each class is small, and the mean difference between classes is large in the space after the projection (Ji and Ye, 2008). Given input data set *D* = {(*x*_1_, *y*_1_),(*x*_2_, *y*_2_), …,(*x*_*n*_, *y*_*n*_)}, *y* ∈ {0,1}, meanwhile, *N*_*j*_(*j* = 0,1) denotes the number of data samples corresponding to the category *j, x* denotes the input and *X*_*j*_(*j* = 0,1) denotes the set of data samples corresponding to the category *j.* The mean vector of the data sample corresponding to class *j* can be expressed as follows:


(21) $$u_{j}=\frac{1}{N_{j}}\sum_{x\in X_{j}}x,j=0,1$$

The covariance matrix of the data sample corresponding to class *j* is denoted as ∑*j*.


(22) $$\sum j=\sum_{x\in X_{j}}\left(x-u_{j}\right){\left(x-u_{j}\right)}^{T},j=0,1$$

Then, the divergence matrix within class is defined as *S*_*w*_.


(23) $$S_{w}=\sum_{x\in X_{0}}\left(x-u_{0}\right){\left(x-u_{0}\right)}^{T}+\sum_{x\in X_{1}}\left(x-u_{1}\right){\left(x-u_{1}\right)}^{T}$$

where *u* denotes mean value.

Simultaneously, the divergence matrix between classes can be described as *S*_*b*_.


(24) $$S_{b}=\left(u_{0}-u_{1}\right){\left(u_{0}-u_{1}\right)}^{T}$$

In this way, the optimization objective is rewritten as follows:


(25) $$\arg\max J\left(w\right)=\frac{w^{T}S_{b}w}{w^{T}S_{w}w}$$

According to (21–25), it can be inferred as follows:


(26) $$w=S_{w}^{-1}\left(u_{0}-u_{1}\right)$$

That is, the optimal projection direction *w* can be determined by calculating the mean and variance of the original two types of samples, and then, the classification is conducted on this basis.

The LR classifier is a probabilistic statistical classification model, which uses a probability score as the predicted value of the dependent variable to evaluate the mutual relation between the dependent variable and the independent variable. Specifically, we utilize the L2 regularization considering the overfitting problem in this study (Chen et al., 2011). The loss function with L2 regularization can be described as follows:


(27) $$\begin{array}{c} J\left(\theta \right)=\frac{1}{m}\sum_{i=1}^{m}\left[-y^{\left(i\right)}\log\left(h_{\theta }\left(x^{\left(i\right)}\right)\right)\right]\\ \;-\frac{1}{m}\sum_{i=1}^{m}\left[\left(1-y^{\left(i\right)}\right)\log\left(1-h_{\theta }\left(x^{\left(i\right)}\right)\right)\right]\\ \;+\frac{\eta }{2m}\sum_{j=1}^{n}\theta_{j}^{2} \end{array}$$

where *h*_θ_(*x*) is defined as:


(28) $$h_{\theta }\left(x\right)=\frac{1}{1+e^{-\theta^{T}x}}$$

Then, we take the derivative concerning (27).


(29) $$\frac{\partial J\left(\theta \right)}{\partial \theta_{j}}=\frac{1}{m}\sum_{i=1}^{m}\left(h_{\theta }\left(x_{i}\right)-y_{i}\right)x_{ij}+\frac{\eta }{m}\theta_{j}$$

where *m* represents the number of data samples, and ηrepresents the regularization coefficient. Finally, the parameters θ_*j*_ corresponding to the minimum loss function are obtained through continuous iteration.

The SVM classifier is to obtain the optimal separating hyperplane in the feature space to maximize the interval between positive and negative data samples on the training set. It is also worth noting that the kernel function adopted in this study is the Gaussian Radial Basis Function kernel (Zhou et al., 2014). The SVM classifier mainly works around the following optimization problems (Cristovao et al., 2022).


(30) $$\begin{array}{c} \min_{w,b}\frac{1}{2}{||w||}^{2}+C\sum_{i=1}^{N}\psi_{i}\\ s.t.y_{i}\left[w^{T}x_{i}+b\right]\geq 1-\psi_{i},\psi_{i}\geq 0 \end{array}$$

The Gaussian kernel is described as follows:


(31) $$K\left(x,x_{i}\right)=\exp\left(-\frac{{||{\text{x-x}}_{i}||}^{2}}{\sigma^{2}}\right)$$

where *C* represents the penalty coefficient, *x*_*i*_ represents the data sample, *y*_*i*_ represents class corresponding to ith data sample, *w* and *b* represent the optimal parameters of the model, *N* represents the number of data, and ψ_*i*_ represents the relaxation variable that corresponds to the ith data sample.

In this study, the leave-one-out cross-validation is employed to avert the overfitting problem of classifiers and overcome the limited number of data, which means that one data sample is employed as the testing set and the remaining samples are employed as the training set. Besides, the parameter adjustment process of the classifier follows the optimal principle.

### Evaluation Criteria

In this study, we obtain features using the MGS-WBC model and VGBN-LM model. To evaluate the validity of obtained features, the most widely used measurements for binary classification problems, such as ACC, F1-Score, and AUC, are adopted based on the confusion matrix (Kam et al., 2020).


(32) $$ACC=\frac{TP+TN}{TP+TN+FP+FN}$$

(33) $$Precision=\frac{TP}{TP+FP}$$

(34) $$Sensitivity=\frac{TP}{TP+FN}$$

(35) $$F1-Score=\frac{2\times Precision\times Sensitivity}{Precision+Sensitivity}$$

**FIGURE 3:**
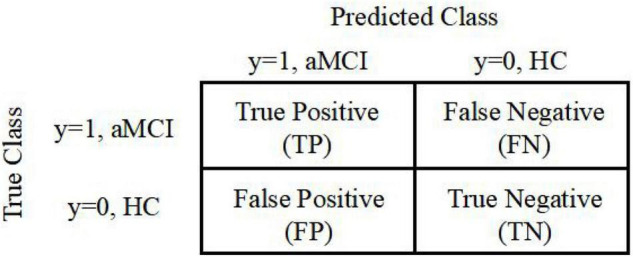
Confusion matrix used to measure the binary classification problems.

True positive (TP), false negative (FN), false positive (FP), and true negative (TN) are defined to calculate the evaluation indicators of classifiers, as is shown in Figure 3.

## Experiment Results and Analysis

In this section, compared with the traditional method, we first analyze the diagnostic performance of aMCI by using the whole-brain structural features obtained from the MGS-WBC model as the input of four classifiers. Then, we analyze the diagnostic performance of aMCI by using the global features obtained from VGBN-LM model and local features obtained from MGS-WBC model as the input of four classifiers compared with the traditional method. In the end, we fuse all features, including whole-brain structural features, local features, and global features, to further improve the diagnostic performance of aMCI. It is notable that the order of the above four classifiers is as follows: NB, LDA, LR, and SVM.

### The Performance Analysis of Whole-Brain Structural Features

**FIGURE 4:**
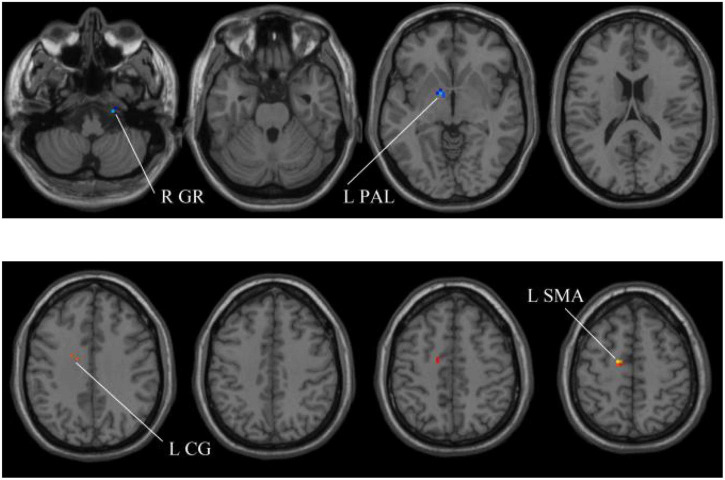
Compared with the HC group, the ReHo in aMCI group exhibits prominent differences based on MGS-WBC^2^. L, left; R, right; GR, gyrus rectus; PAL, pallidum; CG, cingulate gyrus; SMA, supplementary motor area.

**FIGURE 5:**
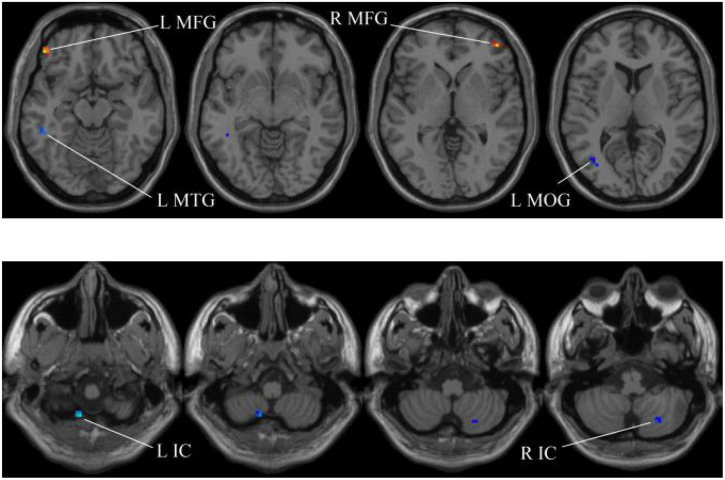
Compared with the HC group, the ReHo in aMCI group exhibits prominent differences based on SSW^1^. L, left; R, right; IC, inferior cerebellum; MTG, middle temporal gyrus; MFG, middle frontal gyrus; MOG, middle occipital gyrus.

In our experiments, the MGS-WBC model is adopted in two whole-brain structures, including ReHo and ALFF, to reduce the data redundancy and extract multiscale features. For the whole-brain structure calculated by ReHo, as shown in Table 1 and Figures 4, 5, we obtain 10 obvious regions of the brain using MGS-WBC model, including the right gyrus rectus (GR), left pallidum (PAL), left cingulate gyrus (CG), left supplementary motor area (SMA), left inferior cerebellum (IC), right IC, left middle temporal gyrus (MTG), left middle frontal gyrus (MFG), right MFG, and left middle occipital gyrus (MOG), while only six obvious brain regions, namely, left IC, right IC, left MTG, left MFG, right MFG, and left MOG, are found using a single significant threshold (two-sample *t*-test, *p* < 0.001). Significantly, each significant region is composed of the activated voxels in the brain. Then, the features extracted from 10 significant regions and six significant regions are separately employed as the input of classifiers, including NB, LDA, LR, and SVM, to evaluate the aMCI’s diagnostic performance. Table 2 presents the diagnostic performance of aMCI using different feature extraction methods of the whole-brain structure (ReHo). The results of MGS-WBC^2^ all exceed 66.67% in the four classifiers. Besides, compared with the single significant threshold in the whole-brain structures (SSW^1^) (Zhang et al., 2015; Yang et al., 2018), the results of MGS-WBC^3^ provide the ACC of 88.06, 89.55, 91.04, and 91.04% in the four classifiers, improving by 4.48, 2.98, 10.44, and 4.47%, respectively. Concurrently, the AUC are 94.12, 96.08, 96.88, and 95.72% in four classifiers, and the increases are 2.24, 1.96, 5.53, and 1.51% compared with SSW^1^. For F1-Score, we provide about 87.88, 89.55, 91.18, and 90.91% in four classifiers, and maximum improvements are 4.30, 2.23, 9.49, and 3.95% compared with SSW^1^.

**TABLE 1:** The extracted clusters using MGS-WBC model after ReHo calculation.

Region	Peak/MNI			*t*-score	Cluster size
	x	y	z		
R GR	21	−18	−45	−2.9046	5
L PAL	−12	0	−3	−2.6854	5
L CG	−15	−6	36	2.3872	5
L SMA	−15	−9	54	3.3572	9
L IC	−12	−63	−60	−4.2059	8
R IC	24	−69	−48	−3.8093	24
L MTG	−51	−39	−12	−4.0141	11
L MFG	−48	42	−15	4.2324	7
R MFG	45	48	0	4.1363	7
L MOG	−45	−66	3	−3.7574	10

*The x, y, and z coordinates are the primary peak locations in the MNI space. Cluster size ≥ 5 voxels in two-sample t-test. L, left; R, right; GR, gyrus rectus; PAL, pallidum,; CG, cingulate gyrus; SMA, supplementary motor area; IC, inferior cerebellum; MTG, middle temporal gyrus; MFG, middle frontal gyrus; MOG, middle occipital gyrus.*

**TABLE 2:** Performance metrics of different classifiers using MGS-WBC model after ReHo calculation.

Classifier	Method	ACC	F1-Score	AUC
NB	SSW^1^	0.8358	0.8358	0.9189
	MGS-WBC^2^	0.6866	0.6667	0.7460
	MGS-WBC^3^	0.8806	0.8788	0.9412
LDA	SSW^1^	0.8657	0.8732	0.9412
	MGS-WBC^2^	0.7164	0.6885	0.7799
	MGS-WBC^3^	0.8955	0.8955	0.9608
LR	SSW^1^	0.8060	0.8169	0.9135
	MGS-WBC^2^	0.7164	0.7077	0.7861
	MGS-WBC^3^	0.9104	0.9118	0.9688
SVM	SSW^1^	0.8657	0.8696	0.9421
	MGS-WBC^2^	0.7612	0.7419	0.7709
	MGS-WBC^3^	0.9104	0.9091	0.9572

*SSW^1^ refers to the single significant threshold (two-sample t-test, p < 0.001) in the whole-brain structures (Zhang et al., 2015; Yang et al., 2018). MGS-WBC^2^ refers to the combination of volatility detection and significant threshold (0.001 < p < 0.05) in the MGS-WBC model. MGS-WBC^3^ refers to the fused features, that is, the final output of MGS-WBC model.*

**FIGURE 6:**
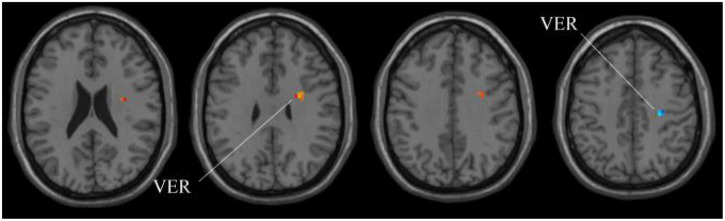
Compared with HC group, the ALFF in aMCI group exhibits prominent differences based on volatility detection in the MGS-WBC model. VER, Vermis.

Next, for the whole-brain structure after ALFF computation, as shown in Table 3 and Figure 6, only two significant regions of the brain corresponding to different locations are found, whereas no significant region exists using SSW^1^. As shown in Table 4, the results of MGS-WBC^2^ or MGS-WBC^3^ provide the ACC of 65.67, 73.13, 71.64, and 67.17% in four classifiers. In addition, we get the AUC of 65.67, 77.72, 77.18, and 75.22% in four classifiers. For F1-Score, we obtain 65.67, 72.73, 69.84, and 60.71% in four classifiers.

**TABLE 3:** The extracted clusters using MGS-WBC model after ALFF calculation.

Region	Peak/MNI			*t*-score	Cluster size
	x	y	z		
VER	27	0	33	2.6341	21
VER	27	−21	45	−2.7783	5

*The x, y, and z coordinates are the primary peak locations in the MNI space. Cluster size ≥ 5 voxels in two-sample t-test. VER, vermis.*

**TABLE 4:** Performance metrics of different classifiers using MGS-WBC model after ALFF calculation.

Classifier	Method	ACC	F1-Score	AUC
NB		0.6567	0.6567	0.6567
LDA	MGS-WBC^2^	0.7313	0.7273	0.7772
LR	/MGS-WBC^3^	0.7164	0.6984	0.7718
SVM		0.6716	0.6071	0.7522

*MGS-WBC^2^ refers to the combination of volatility detection and significant threshold (0.001 < p < 0.05) in MGS-WBC model. MGS-WBC^3^ refers to the fused features, that is, the final output of MGS-WBC model.*

Our goal is to make the average difference between the classes larger and the variance within the classes more stable. Consequently, we develop an MGS-WBC model using multiple significant thresholds and validation detection to generate the fused features of whole-brain structures. Through ablation experiments, it can be inferred that the features of whole-brain structures extracted using MGS-WBC can significantly improve the diagnostic performance of aMCI in typical classifiers compared with the SSW^1^. It indicates that the MGS-WBC model we present is valid, and the model can also effectively remove redundant information from the brain.

### The Performance Analysis of Functional Network Features

In our experiments, to fully excavate the features of the functional network, we develop an MGS-WBC model to extract local features. Meanwhile, we present a VGBN-LM model to extract global features of the functional network and fuse its local features to improve the diagnostic performance of aMCI.

**FIGURE 7:**
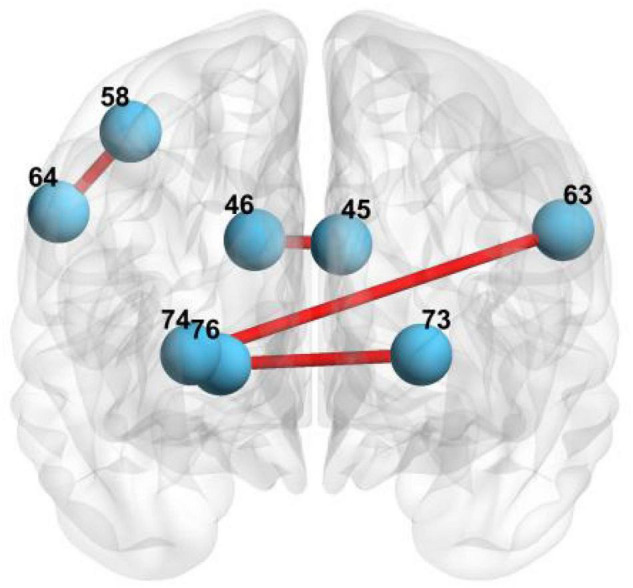
The brain regions involved in local feature selection: 45 represents the left cuneus; 46 represents right cuneus; 73 represents left putamen; 76 represents right pallidum; 74 represents right putamen; 64 represents right supramarginal gyrus; 58 represents postcentral gyrus; and 63 represents left supramarginal gyrus.

First, we select the local features of the functional network using MGS-WBC model to improve the diagnosis performance of aMCI. Our findings elucidate that three pairs of connected brain regions with notable differences are found using MGS-WBC^2^, including (45, 46), (73, 76), and (74, 76), as can be seen from Table 5. Also, two pairs of connected brain regions, such as (64, 58) and (63, 74), are found using SSW^1^. The brain regions involved in the local feature selection are shown in Figure 7, where the light blue ball denotes the selected brain regions, and the red line indicates that the two selected brains are connected. Notably, the number in Figure 7 and Table 6 denotes the brain regions segmented using the AAL template. The diagnosis performance of aMCI using local features resulted from the MGS-WBC model is sorted in Table 5 compared with SSF^1^. When the features generated from MGS-WBC^2^ are used as the input of the four classifiers, including NB, LDA, LR, and SVM, the classification metrics ranged from 55.38 to 69.61%. Meanwhile, the results elucidate that except for LDA, each of NB, LR, and SVM classifiers makes large improvements using MGS-WBC^3^ compared with SSF^1^. Although the ACC and F1-Score of LDA decreased by 1.49 and 0.24%, respectively, the AUC value increased by 5.35%.

**TABLE 5:** Performance metrics of local features using MGS-WBC model.

Classifier	Method	ACC	F1-Score	AUC
NB	SSF^1^	0.7313	0.7188	0.7496
	MGS-WBC^2^	0.5672	0.5538	0.6346
	MGS-WBC^3^	0.7463	0.7463	0.8048
LDA	SSF^1^	0.7313	0.7188	0.7531
	MGS-WBC^2^	0.6269	0.6032	0.6854
	MGS-WBC^3^	0.7164	0.7164	0.8066
LR	SSF^1^	0.6567	0.5490	0.7308
	MGS-WBC^2^	0.6119	0.5938	0.6961
	MGS-WBC^3^	0.7015	0.6667	0.7995
SVM	SSF^1^	0.7612	0.7576	0.7647
	MGS-WBC^2^	0.6418	0.5862	0.6943
	MGS-WBC^3^	0.7761	0.7826	0.8324

*SSF^1^ refers to the single significant threshold (p < 0.001) in the functional network (Zhang et al., 2015; Yang et al., 2018). MGS-WBC^2^ refers to the combination of volatility detection and significant threshold (0.001 < p < 0.05) in MGS-WBC model. MGS-WBC^3^ refers to the fused features, that is, the final output of MGS-WBC model.*

**TABLE 6:** Local feature selection of functional network using MGS-WBC model.

Connected regions	Group	Volatility detection	*p*-value
(45, 46)	aMCI	0.2611	0.0157
	HC	0.3063	0.0462
(73, 76)	aMCI	0.6176	0.0335
	HC	0.5038	0.0335
(74, 76)	aMCI	0.5172	0.0133
	HC	0.3487	0.0133
(64, 58)	aMCI and HC	–	0.0006
(63, 74)	aMCI and HC	–	0.0006

*45 represents the left cuneus; 46 represents right cuneus; 73 represents left putamen; 76 represents right pallidum; 74 represents right putamen; 64 represents right supramarginal gyrus; 58 represents postcentral gyrus; and 63 represents left supramarginal gyrus.*

We further develop a VGBN-LM model to extract global features of the functional network, including left MFG (*p* = 0.035, two-sample *t*-test), left hippocampus (*p* = 0.030, two-sample *t*-test), right hippocampus (*p* = 0.038, two-sample *t*-test), left amygdala (*p* = 0.009, two-sample *t*-test), and right amygdala (*p* = 0.013, two-sample *t*-test), and the global features, are then used as the input of classifiers to evaluate the validity of the extracted features. As summarized in Table 7, the results reveal that the ACC, F1-Score, and AUC in the four classifiers can achieve more than 61% using VGBN-LM. Moreover, the results of VGBN-LM can provide the ACC of 64.18, 62.69, 65.67, and 61.19% in four classifiers, and maximum improvements are 19.40, 5.97, 13.43, and 14.92% compared with VGBN-LM^1^. Also, compared to VGBN-LM^1^, the AUCs of the four classifiers are 69.96, 61.85, 69.79, and 67.29%, and the growth rates are 18.80 30.48, 16.49, and 27.63%. For F1-Score, we provide about 64.18, 62.69, 65.67, and 61.19% in four classifiers, increasing by 28.23, 11.31, 15.67, and 19.11% compared with VGBN-LM^1^. Furthermore, we reduce the number of features per data sample from 4,005 ($\frac{8100-90}{2}$) to 5 using VGBN-LM.

**TABLE 7:** Global features analysis using VGBN-LM model.

Classifier	Method	ACC	F1-Score	AUC
NB	VGBN-LM^1^	0.4478	0.3934	0.5116
	VGBN-LM	0.6418	0.6757	0.6996
LDA	VGBN-LM^1^	0.5672	0.5246	0.3137
	VGBN-LM	0.6269	0.6377	0.6185
LR	VGBN-LM^1^	0.5224	0.5000	0.5330
	VGBN-LM	0.6567	0.6567	0.6979
SVM	VGBN-LM^1^	0.4627	0.4375	0.3966
	VGBN-LM	0.6119	0.6286	0.6729

*VGBN-LM^1^ refers to VGBN-LM model while without using two-sample t-test (p < 0.05). VGBN-LM refers to the method we propose in the “Vector Generation for Brain Networks Based on Laplacian Matrix (VGBN-LM)” Section.*

Then, we fuse the multiscale features of functional network, including local features and global features, to further improve the diagnostic performance of aMCI. The local features of functional network are obtained using SSF^1^ and MGS-WBC^2^. As summarized in Table 8, using MGS-VGBN^1^, each of NB, LDA, LR, and SVM classifiers has a large value in ACC, F1-Score, and AUC, and its values ranged from 65.45 to 80.57%. Specifically, the results of MGS-VGBN^2^ provide the ACC of 76.12, 77.61, 77.61, and 79.10%, and the growth rates are 2.99, 4.48, 11.94, and 2.98% compared with SSF^1^. In addition, we obtain the AUC of 81.64, 81.19, 83.42, and 85.20% in four classifiers, and the improvements are 6.68, 5.88, 10.34, and 8.73% compared with SSF^1^. For F1-Score, we provide about 76.47, 77.61, 76.92, and 78.79% in four classifiers, and the improvements are 4.59, 5.73, 22.02, and 3.03% compared with SSF^1^.

**TABLE 8:** Performance metrics of local and global features of functional network.

Classifier	Method	ACC	F1-Score	AUC
NB	SSF^1^	0.7313	0.7188	0.7496
	MGS-VGBN^1^	0.7313	0.7353	0.7772
	MGS-VGBN^2^	0.7612	0.7647	0.8164
LDA	SSF^1^	0.7313	0.7188	0.7531
	MGS-VGBN^1^	0.7463	0.7536	0.7763
	MGS-VGBN^2^	0.7761	0.7761	0.8119
LR	SSF^1^	0.6567	0.5490	0.7308
	MGS-VGBN^1^	0.7164	0.6545	0.7879
	MGS-VGBN^2^	0.7761	0.7692	0.8342
SVM	SSF^1^	0.7612	0.7576	0.7647
	MGS-VGBN^1^	0.7761	0.7761	0.8057
	MGS-VGBN^2^	0.7910	0.7879	0.8520

*SSF^1^ refers to the single significant threshold (p < 0.001) in the functional network (Zhang et al., 2015; Yang et al., 2018). MGS-VGBN^1^ refers to local features generated from volatility detection of MGS-WBC model and global features generated from VGBN-LM model in functional network. MGS-VGBN^2^ refers to the fused features of functional network.*

Regarding limited and high-dimensional data, we present a VGBN-LM model that excavates the global features of the functional network and fuses local features obtained by the MGS-WBC model. By ablation experiments, multiscale features of functional network we propose can greatly improve the diagnostic performance of aMCI compared to SSF^1^, indicating that the global features we propose can play a certain role in the intelligent diagnosis of aMCI. Besides, the features extracted by this study can accurately locate the significant regions in the brain, which is convenient for doctors to conduct further studies.

### The Performance Analysis of Fused Features

In our experiments, we mainly fuse the multiscale features, including whole-brain structural features and features of functional network, to improve the diagnostic performance of aMCI.

As summarized in Table 9, compared with the single significant threshold (*p* < 0.001) in the whole-brain structures and function network (SSWF^1^) (Zhang et al., 2015; Yang et al., 2018), the ACC of NB, LDA, LR, and SVM classifiers are about 89.55, 91.04, 92.54, and 94.03%, increasing by 1.49, 1.49, 1.50, and 4.48%, respectively. Besides, this study provides the AUC of 95.63, 92.69, 97.59, and 97.33% in the four classifiers, increasing by 2.49, 1.96, 2.67, and 2.23%, respectively. For F1-Score, this study provides about 76.47, 77.61, 76.92, and 78.79% in the four classifiers, increasing by 1.67, 1.05, 1.64, and 4.26%, respectively.

**TABLE 9:** The fused features analysis.

Classifier	Method	ACC	F1-Score	AUC
NB	SSWF^1^	0.8806	0.8788	0.9314
	FUSE	0.8955	0.8955	0.9563
LDA	SSWF^1^	0.8955	0.8986	0.9465
	FUSE	0.9104	0.9091	0.9269
LR	SSWF^1^	0.9104	0.9091	0.9492
	FUSE	0.9254	0.9254	0.9759
SVM	SSWF^1^	0.8955	0.8986	0.9510
	FUSE	0.9403	0.9412	0.9733

*SSWF^1^ refers to the single significant threshold (p < 0.001) in the whole-brain structures and function network (Zhang et al., 2015; Yang et al., 2018). FUSE refers to the fused features obtained from MGS-WBC and VGBN-LM models in the whole-bran structures and functional network.*

Over the years, the extraction of biomarkers of aMCI based on rs-fMRI data has been reported by numerous studies (Yang et al., 2018, 2021), yet very few studies about multiscale feature extraction of whole-brain structures and functional network due to limited data, and how to use machine learning methods to verify the effectiveness of the features. Furthermore, a robust and reliable system for aMCI detection is conducive to timely detecting and screening patients at a high risk of AD. Based on this, we extract multiscale features according to the characteristics of the brain and develop the framework of the diagnostic system. Our proposed models, including MGS-WBC model and VGBN-LM model, outperform the traditional approaches (SSWF^1^) (Zhang et al., 2015; Yang et al., 2018) in this study. It turns out that the fused features obtained from the MGS-WBC model and VGBN-LM model are more important than the traditional single-scale features in terms of the diagnosis of aMCI. Furthermore, our feature selection methods will make the diagnosis of aMCI more accurate and reliable, providing novel insights for the extraction of discriminative neuroimaging features.

## Conclusion

In this study, we present a system for the diagnosis of aMCI. We first develop an MGS-WBC model to extract discriminative features of whole-brain structures and functional networks. Then, we propose a VGBN-LM model that excavates the global features of the functional network and fuses its local features, thus overcoming the low availability of the functional network features. Finally, we fuse all the features generated from the MGS-WBC model and VGBN-LM model to further improve the diagnostic performance of aMCI. The results demonstrate that this study outperforms the traditional method. In conclusion, the proposed feature extraction methods can be utilized to detect other similar neurological diseases of the brain, providing new insights for the intelligent construction of the CAD system. The future study contains applying our approaches to other brain diseases and verifying the robustness of the system. Besides, more data will be collected so that deep learning models can be used for accurate classification.

## Data Availability Statement

Publicly available datasets were analyzed in this study. This data can be found here: http://adni.loni.usc.edu/.

## Ethics Statement

Ethical review and approval was not required for the study on human participants in accordance with the local legislation and institutional requirements. Written informed consent for participation was not required for this study in accordance with the national legislation and the institutional requirements. Ethical review and approval was not required for the animal study because publicly available datasets were analyzed in this study. This data can be found here: http://adni.loni.usc.edu/. Written informed consent was obtained from the individual(s) for the publication of any potentially identifiable images or data included in this article.

## Author Contributions

CC was responsible for writing the manuscript and doing the experiments. CY and EC instructed the experiments. JC was responsible for drawing pictures in the manuscript. All authors contributed to the article and approved the submitted version.

## Conflict of Interest

The authors declare that the research was conducted in the absence of any commercial or financial relationships that could be construed as a potential conflict of interest.

## Publisher’s Note

All claims expressed in this article are solely those of the authors and do not necessarily represent those of their affiliated organizations, or those of the publisher, the editors and the reviewers. Any product that may be evaluated in this article, or claim that may be made by its manufacturer, is not guaranteed or endorsed by the publisher.

## Footnotes

1
http://adni.loni.usc.edu/

2
http://restfmri.net/forum/RESTplusV1.2

3
http://www.mathworks.com/products/matlab/

4
https://www.fil.ion.ucl.ac.uk/spm/software/spm12/
